# The tempos of performance

**DOI:** 10.1016/j.copsyc.2019.06.003

**Published:** 2019-10

**Authors:** Nir Shalev, Anna-Katharina R Bauer, Anna C Nobre

**Affiliations:** Department of Experimental Psychology, Oxford Centre for Human Brain Activity, Department of Psychiatry, and Wellcome Centre for Integrative Neuroimaging, University of Oxford, United Kingdom

## Abstract

Human performance fluctuates over time. Rather than random, the complex time course of variation reflects, among other factors, influences from regular periodic processes operating at multiple time scales. In this review, we consider evidence for how our performance ebbs and flows over fractions of seconds as we engage with sensory objects, over minutes as we perform tasks, and over hours according to homeostatic factors. We propose that rhythms of performance at these multiple tempos arise from the interplay among three sources of influence: intrinsic fluctuations in brain activity, periodicity of external stimulation, and the anticipation of the temporal structure of external stimulation by the brain.

## Highlights

• Performance fluctuates at multiple timescales relevant for cognitive ‘events’ embedded in ‘tasks’ and homeostatic cycles.
• Performance is shaped by intrinsic dynamics, external stimulation, and anticipation mechanisms.
• Future studies should focus on testing interactions among multiple timescales and temporal factors.

**Current Opinion in Psychology** 2019, **29**:254–260

This review comes from a themed issue on **Attention and perception**

Edited by **Sarah Shomstein, Andrew Leber** and **Joy Geng**

For a complete overview see the Issue and the Editorial

Available online 18th June 2019

**https://doi.org/10.1016/j.copsyc.2019.06.003**

2352-250X/© 2019 The Authors. Published by Elsevier Ltd. This is an open access article under the CC BY license (http://creativecommons.org/licenses/by/4.0/).

Behavioural performance varies over time. It is increasingly recognised that the variability is not simply random, but also reflects systematic fluctuations at multiple timescales. Fluctuations are observed within single episodes of task performance, over fractions of seconds, possibly reflecting how the intrinsic dynamics of neural signalling affect the interface with sensory objects [1]. At the longer timescales of minutes, periodic changes are observed in performance in psychophysical tasks [2] and in task engagement [3]. Beyond hours, periodic changes in performance occur over ultradian [4], diurnal, and circadian cycles [5]; as well as in weekly [6], menstrual [7], and seasonal [8] rhythms.

**Figure 1:**
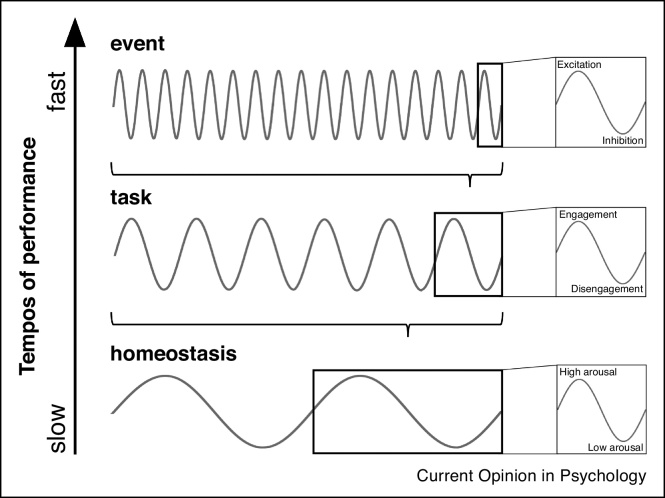
A schematic overview of different tempos for fluctuating performance, ranging from the shortest, event-related timescales in milliseconds to hours and days. Recognising that there may be a richer continuum of time scales, we represent three types of tempos. At the fastest time scale, rhythms may relate to fluctuating excitability within brain areas and in neural circuits. At the intermediate time scale, rhythms may relate to changes in arousal and different levels of engagement during sustained task performance over minutes to hours. At the slowest time scales, performance fluctuations may be linked to homeostatic factors determining energy levels of the organism over the day.

Whereas the significance of periodic fluctuations in performance begins to be acknowledged within given temporal scales, a more integrative consideration of variation over multiple time scales is lacking. In this review, we highlight how performance ebbs and flows over multiple tempos, and invite consideration of how the various rhythms may be organised and inter-related. To aid the organisation of the discourse, we distinguish among fast ‘event’ tempos (>1 Hz) that are meaningful for a single cognitive event in a trial (e.g. discriminating a stimulus or performing an action), slower ‘task’ tempos (seconds to minutes) that are relevant for extended performance within a cognitive task, and even slower ‘homeostatic’ tempos (hours to days) that dictate the energetic state of the organism at a given time. In Figure 1, we present a schematic overview of the proposed timescales and their corresponding constructs. We propose that it is essential to consider three factors that may contribute to periodic fluctuations in performance: (1) rhythmic fluctuations that are *intrinsic* to processing units and systems within the brain, (2) periodicity of external stimulation, and (3) the ability of the brain to learn *external* temporal structures and generate *anticipatory mechanisms* to guide selection and prioritisation of relevant events in the service of adaptive behaviour.

## Event tempos

Fast periodicity of perceptual systems was proposed on theoretical grounds before it was directly observed [9, see also Ref. 10]. Periodic and discrete sampling of sensory information, or the ongoing ‘parsing’ of continuous sensory input, was proposed to be advantageous for enabling multiplexing of information processing and for providing time stamps for integrating and sequencing information [9]. Behaviourally, rapid performance modulations (>1 Hz) have been observed for making perceptual judgments about visual features, such as motion, depth, or colour [11].

### Intrinsic brain fluctuations

Studies combining measurements of behavioural performance and neural activity have revealed that the intrinsic rhythmic fluctuations of brain activity may contribute to the periodicity of behavioural performance. Fluctuations in behavioural performance at timescales above 1 Hz correlate with the ebb and flow of intrinsic neural oscillations^2^ [12, 13, 14]. Supporting the notion of periodic sensory sampling, studies have shown that variability in perceptual performance co-varies with intrinsic brain rhythms, especially in the theta (∼4–8 Hz) and alpha (∼8–12 Hz) frequency bands. For instance, visual target detection of near-threshold stimuli fluctuates in phase with neural oscillations in the alpha band [12,13,15]. So far, most studies investigating perceptual fluctuations in relation to the phase of intrinsic brain oscillations have been in the visual modality. However, a recent paper suggests that oscillations in behavioural performance also exist in the auditory domain in the theta frequency range [16].

In addition, studies using ‘reset events’ provide evidence for periodic sensory sampling in the theta frequency range [14,17, 18, 19, 20]. Reset events can be generated by a transient salient event, thought to reset the phase of ongoing intrinsic neural oscillations so that subsequent oscillations become phase-locked to the reset event. Through the presentation of response-relevant stimuli at various intervals after such a reset event, the unfolding of behavioural performance fluctuations can be directly measured. For example, in a spatial attention task with two behaviourally relevant locations, a reset event was used to investigate the periodic sampling of each location [17]. This approach assumed that the reset event captures attention, thus prioritising sampling of its location. Following the reset event, visual target detection accuracy for each location fluctuated at a 4-Hz rate, while performance between the two locations was in antiphase. Periodic sensory sampling of this type has also been found to be triggered by reset events caused by auditory stimuli [20] and movement onsets [21].

Rhythmic performance fluctuations have been predominantly reported for the theta and alpha frequency bands. While we are not aware of studies showing direct evidence for sensory performance fluctuations in the gamma frequency range (>30 Hz), there is evidence that the phase of delta (0.5–4 Hz) and theta rhythms modulates gamma and alpha band activity and neuronal firing [22, 23, 24, 25]. The informational content of neural processing is thought to be carried in these high-frequency signals. Therefore, their regulation by the slower delta and theta oscillations supports the early theoretical proposals of periodic sampling and processing of sensory information [9,10]. Such a mechanism could enable the pick-up and relay of information within local neuronal ensembles to be quantised and paced by capitalising on the slower intrinsic oscillations reflecting the circuit-level dynamics of the networks in which they are embedded.

### Entrainment to periodic stimulation

Intrinsic brain rhythms can contribute to fluctuating patterns of performance even in the absence of temporally structured external stimulation [1,12, 13, 14, 15]. However, additionally, performance is also highly sensitive to periodicities in external stimulation [26]. Many natural stimuli that guide behaviour, such as speech, music, or footsteps, follow a regular rhythm, occurring predominantly between 0.5 and 4 Hz. The influence of periodic, rhythmic stimulation on performance was demonstrated in a series of psychophysical experiments in the auditory domain. Perceptual identification and discrimination of auditory tones were most accurate when stimuli occurred in phase with the preceding rhythmic stimulus train [27, but see Ref. 28].

In principle, two different types of mechanisms can account for performance benefits in the context of periodic external stimulation. The simplest is a mechanism of reactive ‘entrainment’ by which intrinsic neural oscillations are reactively and automatically reset and paced by external events [29]. The alternative is a mechanism of proactive anticipation, by which the brain learns about the periodicities in external stimulation and uses top-down signals to prepare sensory systems for relevant upcoming sensory events [30,31].

Entrainment mechanisms were invoked to explain performance benefits in rhythmic stimulation contexts on theoretical and computational grounds in the ‘dynamic attending theory (DAT)’ by Jones and colleagues [27,32]. Neural recordings later confirmed that neural oscillations do entrain to rhythmic stimulation in the delta and theta ranges, thereby providing a plausible physiological basis for DAT [29,33]; see also Ref. [34]. When presented with rhythmic auditory stimulation, fluctuations in behavioural performance are dependent on the phase of the entrained neural oscillations [35], and behavioural modulations can be observed even in the absence of abrupt onsets in the entraining sequence [36,37]. While many studies have focused on the auditory modality, stimulus-driven fluctuations of performance have also been noted in the visual modality, particularly in the alpha range [38,39].

### Proactive temporal anticipation

In rhythmic contexts, separating effects attributed to slavish stimulus-driven entrainment versus proactive anticipation is difficult if not impossible [40]. Many different types of anticipation mechanisms have been described [41]. In addition to rhythmic anticipation [27], anticipation can be related to learned temporal associations between individual events [42], sequences of events [31,43], and temporal conditional probability [44,45]. These multiple temporal structures can combine and occasionally interact [46, 47, 48].

It is likely that in rhythmic contexts both entrainment and temporal anticipation occur, and that these interact further with other sources of top-down attention-related signals that guide prioritisation and selection of relevant stimuli. For example, when monkeys were presented with interleaved rhythmic auditory and visual stimulation, performance fluctuations and neural oscillatory activity were dependent on which stimulus modality was relevant for performance [29].

## Task tempos

Slower oscillations in the range of seconds reflect processes that affect sustained task performance. For example, studies of vigilance examine the capacity to maintain an adequate state of arousal and focus to detect occasional targets within repetitive and non-engaging tasks over minutes or hours [3,49,50]. Whereas traditionally studies of vigilance have investigated the decrement of performance over time [49,51, 52, 53], some have emphasised the waxing and waning of performance [50,54]. Potentially, clinical and neurotypical populations can be distinguished based on performance rhythms. For example, studies have shown that children diagnosed with ADHD manifest a unique oscillatory pattern of periodic drops in accuracy every 20–30 s [55]. It was also speculated that children with ADHD exhibit atypical rhythmic fluctuations in the ‘default-mode network’ which normally fluctuates between 0.01 and 0.1 Hz [56]. A different study showed that individuals with ADHD have diminished ability to benefit from rhythmic patterns in a continuous performance task compared to neurotypical individuals [57].

### Intrinsic brain fluctuations

Empirical findings concerning occasional disengagements from continuous performance tasks [54] are normally attributed to a gradual inability to sustain attention [58]. However, intrinsic oscillatory properties of performance may also contribute. Task rhythms may partly reflect ebbing and flowing of different brain networks [59,60]. The existence of functionally significant slow oscillations ranging between 0.01 and 0.2 Hz is supported by modelling data [61], local field-potential recordings in monkeys [62], and human electrophysiology [63]. These rhythms are thought to result in periodic changes in psychophysical performance parameters [59,64]. They are associated with the clustering of performance levels in cognitive tasks, for example, when detection rates on consecutive trials are auto correlated for time lags longer than 100 s [64]. At the neural level, they are thought to represent a slow cyclic modulation of gross cortical excitability [65].

Interestingly, these oscillations below 1 Hz (infraslow) can also interact with faster rhythms. Empirical evidence suggests that the phase of infraslow brain oscillations correlates with the amplitude of faster rhythms (1–40 Hz) [64]. Accordingly, it has been proposed that the ongoing intrinsic infraslow fluctuations between 0.01 and 0.1 Hz and the faster oscillations (between 1 and 40 Hz) nested therein may account for the typical correlation in performance among successive trials in behavioural tasks, creating non-random clustering of performance patterns over time, with variability increasing at longer time-scales [64]. When describing the time series of psychophysical performance over minutes, behavioural data exhibit fractal patterns and power-law autocorrelations [66]. Such dynamics that are characterised by patterns of hierarchical self-similarities at multiple time-scales are typical of ‘scale-free dynamics’, or 1/f distributions, which seem to be a common motif of both behavioural performance patterns [2,67] and brain activity [63, 64, 65].

### Entrainment to periodic stimulation

Some evidence suggests that entrainment to external periodic stimulation is not confined to the timescale of milliseconds. Using intracellular recordings in animals, researchers have identified non-lemniscal auditory neurons in the thalamus with spontaneous up/down transitions at random intervals, which can become entrained to rhythmic stimulation occurring between 3 and 12 s [68]. However, we are not familiar with comparable findings in the human literature showing entrainment at such slow rhythms.

### Proactive temporal anticipation

At the slower rhythm of sustained task performance, temporal anticipation of target events may interact with the regulation of arousal. Changes in tonic arousal during task performance have been associated with changes in uncertainty levels [69, 70, 71]. The potential interaction between external stimulus rhythms and arousal is often discussed in the sustained-performance literature. To some extent, the very first experimental task manipulating vigilance relied on rhythmic stimulation [49]. Many other task designs that followed the traditional vigilance (and later: sustained attention) research also presented stimuli in fixed or regular rhythmic intervals [50,72,73]. In our lab, we have recently observed that presenting target events within a predictable, rhythmic temporal structure leads to a periodic modulation of pupil size in preparation for stimulus onset, alongside a reduction in the overall arousal as indexed by tonic changes in pupil size [74]. In contrast, temporally unpredictable targets are associated with a continuous state of high arousal. Our results suggest that traditional explanations of changes in arousal caused by habituation of the neural response to repetitive stimulation [75] or by ordinal predictability [76] may be insufficient, as they do not account for the effects specifically attributable to the temporal structure of the task.

## Homeostatic tempos

### Intrinsic brain fluctuations

When moving from minutes to hours, further intrinsic rhythmic components influence performance. For example, researchers have shown that fluctuations in arousal can occur independently of circadian cycles based on a dopaminergic ultradian oscillator [77]. Electrophysiological studies support the notion of such slow rhythmic fluctuations. For example, researchers have identified two separate components of arousal in broadband EEG, one fluctuates in cycles of ∼100 min, and is related to changes in vigilance; the other fluctuates between 3 and 8 h, and represents variations in wakefulness levels [78]. The functional significance of ultradian rhythms of arousal was demonstrated in a study tracking the latency and amplitude of event-related potentials showing reliable rhythmic fluctuations over hours [79]. Similarly, it was shown that the pattern of performance decrement during a prolonged vigilance task is mirrored by a decrease in the trial-by-trial consistency of the neural response in the theta phase (3–7 Hz), providing further evidence for the association between task and event rhythms [80]. Thus, although changes in arousal are often discussed in the context of habituation resulting from repetitive stimulation [75], it is also affected by very slow intrinsic rhythmic components.

### Entrainment to periodic stimulation

Whereas the literature has not yet highlighted the effect of infraslow periodic stimulation on performance, a clear stimulus-driven component is recognised to affect performance fluctuations related to ultraslow circadian rhythms. In the ‘forced desynchrony’ experimental protocol, participants are placed in artificial light/dark cycles for varying durations [81]. Using this approach, researchers have shown that fluctuations in body temperature and heart rate are determined by both external stimulation and homeostatic rhythmic components [82]. In turn, periodic changes to body temperature are causally related to fluctuations in cognitive performance [83]. Furthermore, models of dynamic changes in human performance within the time scale of days discuss the circadian processes that periodically determine both the sleep drive and waking alertness, with the latter being closely associated with overall performance [84].

A different source of a periodic stimulation which potentially interacts with brain oscillations occurs through the interaction with other physiological processes in the body. A recent study has demonstrated the phase-amplitude coupling between alpha activity in the brain and an infra-slow gastric basal rhythm (∼0.05 Hz) generated by the stomach [85]. The study has shown that approximately 8% of the variance in alpha activity can be explained by the oscillations generated by the gut (for a recent review on how visceral signals shape neural activity see Ref. [86]). However, it is still unclear whether such periodic stimulation is directly associated with changes in behaviour.

## Summary and future directions

**Figure 2:**
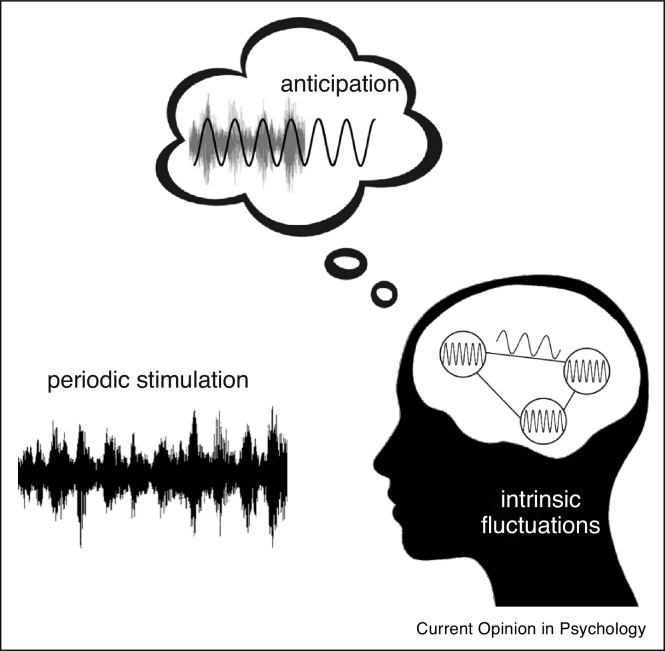
We propose that three separate factors contribute to structuring performance over time. We provide an example in which a speech stimulus contains regular periodic modulations (periodic stimulation). This temporally structured external stimulus interacts with the intrinsic fluctuations of ongoing brain activity, including high-frequency oscillations related to neuronal activity in local circuits and lower-frequency oscillations related to dynamics in networks of interconnected regions. Furthermore, the brain learns about the temporal regularities in the external stimulus and is able to use prospective signals to anticipate the occurrence of relevant events. The anticipation function depicted in the thought bubble shows the pickup of the periodicity in the external stimulus to project the likely pattern of future input.

Rhythmicity is a hallmark of the brain [1] and its environment, and characterizes many aspects of cognition and behaviour [87]. Observations of the rhythmic facilitation of behaviour appeared in the earliest days of cognitive research [26], and have since been substantiated by neuroscientific evidence [29]. In this review we have considered rhythms affecting behavioural performance at multiple timescales — from single events, to sustained tasks, to throughout the day. We have suggested that multiple factors play a role in structuring performance over time — fluctuations in intrinsic brain and homeostatic mechanisms, reactive entrainment to external period stimulation, and proactive anticipation of the temporal structure of events (see Figure 2).

The exciting topic for future research will be to understand whether and how the factors influencing performance at these various tempos interact. Here we noted a few examples available so far, such as putative interactions between task rhythms and event rhythms that could be mediated by the effects of arousal on faster attention-related dynamics through its change in cortical signal-to-noise ratio [69,71], and the interplay between even slower homeostatic functions and faster signatures of brain activity [68]. However, most of the fun work is still ahead, and results are likely to reveal interesting fundamental principles about the coordination of brain activity and behavioural output. Headway will depend on us broadening the temporal focus of our experimental tasks. Rather than just taking performance measures during single trials as isolated events, it will be fruitful to move to dynamic and extended task contexts, to measure brain and homeostatic activity at multiple time scales, to vary the temporal regularity and pace of stimulation, and to manipulate the predictability of temporal structures.

## Conflict of interest statement

Nothing declared.

## Footnotes

2 Neural oscillations reflect fluctuations between high and low excitability states in neuronal circuits, emerging from the temporal kinetics of the voltage-gated channels involved in neuronal excitability and the complexity of the circuit [1]. For example, high-frequency gamma oscillations in cortex may arise from local microcircuits interconnecting excitatory pyramidal neurons and inhibitory interneurons. Slower, alpha-band oscillations may involve circuits joining neuronal ensembles in different regions, such as thalamic and cortical regions.
